# Supplementary material for: Arabidopsis guard cell chloroplasts import cytosolic ATP for starch turnover and stomatal opening
Source: Nat Commun. 2022 Feb 3;13:652. doi: 10.1038/s41467-022-28263-2 (PMC8814037; doi:10.1038/s41467-022-28263-2)
Supplement: Supplementary file 1 — Supplementary Information [file 41467_2022_28263_MOESM1_ESM.pdf]

Supplementary Information for

Arabidopsis guard cell chloroplasts import cytosolic ATP for starch turnover and stomatal opening

Lim *et al.*

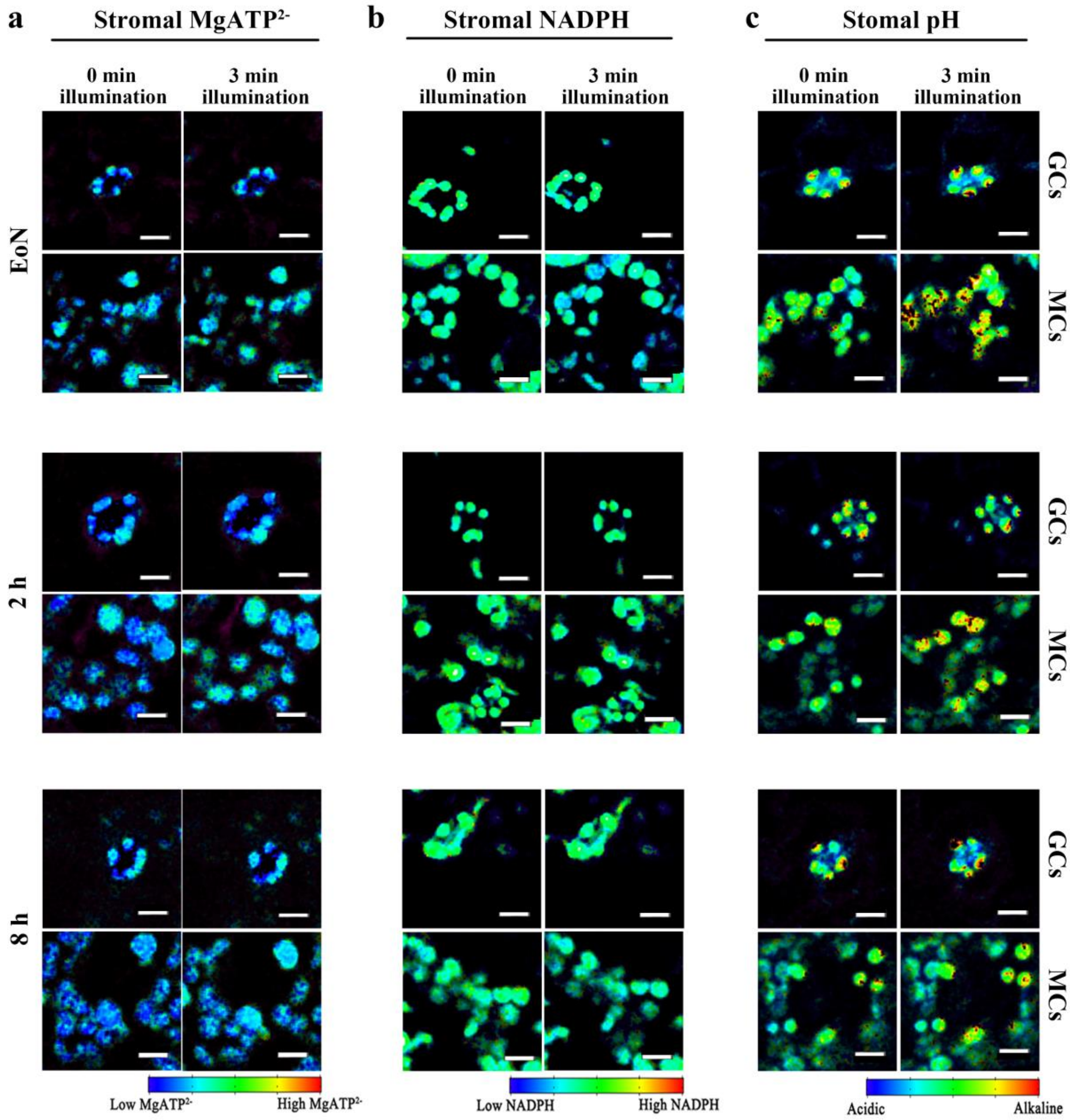

Supplementary Figure 1. Light responses of stromal $MgATP^{2-}$, NADPH and pH sensors in mesophyll cells and guard cells. Representative confocal microscopy ratiometric images of mesophyll cell chloroplasts (MCCs) and guard cells chloroplasts (GCCs) of 20- to 22-day-old plants before and after 3 min of illumination at 216 μmol m^{-2} s^{-1}. **a**, Stromal AT1.03, **b**, Stromal iNAP4 and **c**, Stromal cpYFP signals were collected at different time points (EoN, 2 h and 8 h into the day). **EoN**, End of night. Scale bars, 10 μm. This experiment was repeated two times independently. Ratios of the Venus/CFP, raw iNAP4 ratio and cpYFP ratio images are represented in pseudo colour.

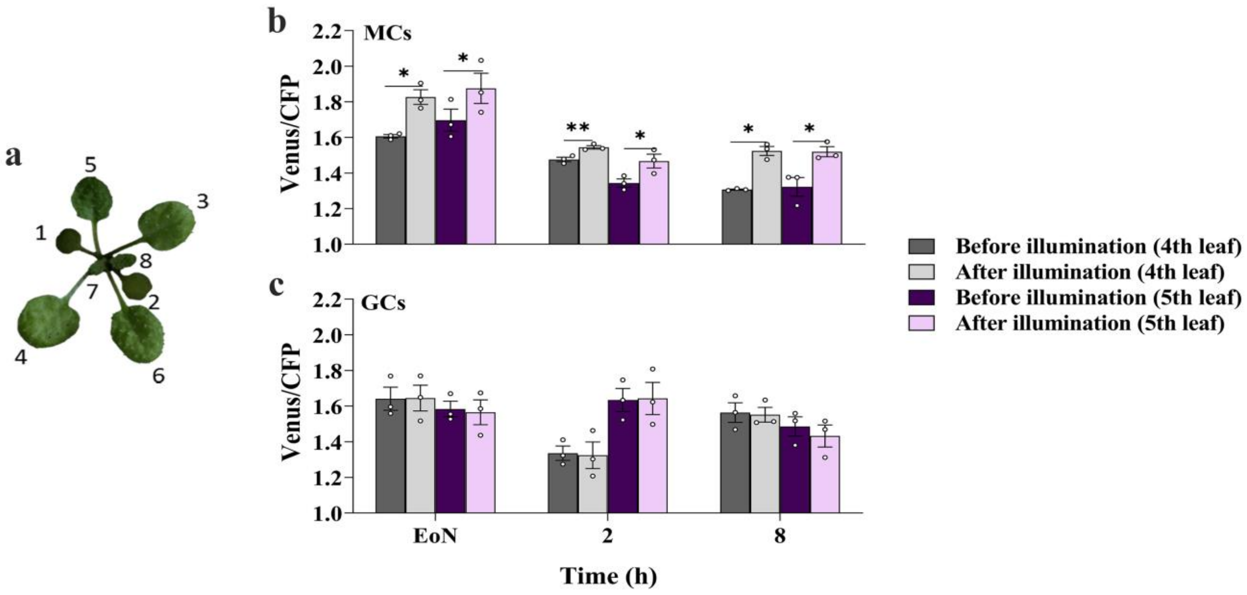

Supplementary Figure 2. Stromal ATP generation in leaves of different developmental stages.
a, Photograph of a representative Arabidopsis 21-day-old plant. Signals of the stromal ATP sensor AT1.03 in mesophyll cells (MCs) and guard cells (GCs) of young (5th leaf) and mature leaves (4th leaf) collected at the EoN and after 2 h or 8 h of illumination. A fluence rate of 216 μmol m^{-2} s^{-1} white light was applied for 3 min after the first image was captured at the indicated time points. **b,** AT1.03 signal from the plastid stroma in MCs, (p-values of panel b (4th leaf MCs): EoN = 2.8 x 10^{-3}, 2 h = 0.006, and 8 h = 0.016; 5th leaf MCs : EoN = 0.017, 2 h = 0.018, and 8 h = 0.043), and **c**, AT1.03 signal from the plastid stroma in GCs. **EoN**, End of night. Asterisks indicate significant statistical differences (*P < 0.05, **P < 0.01) before and after 3 min of illumination, as determined by paired *t*-test, two-tailed (n = 3; error bars ± SEM). Source data are provided as a source data file.

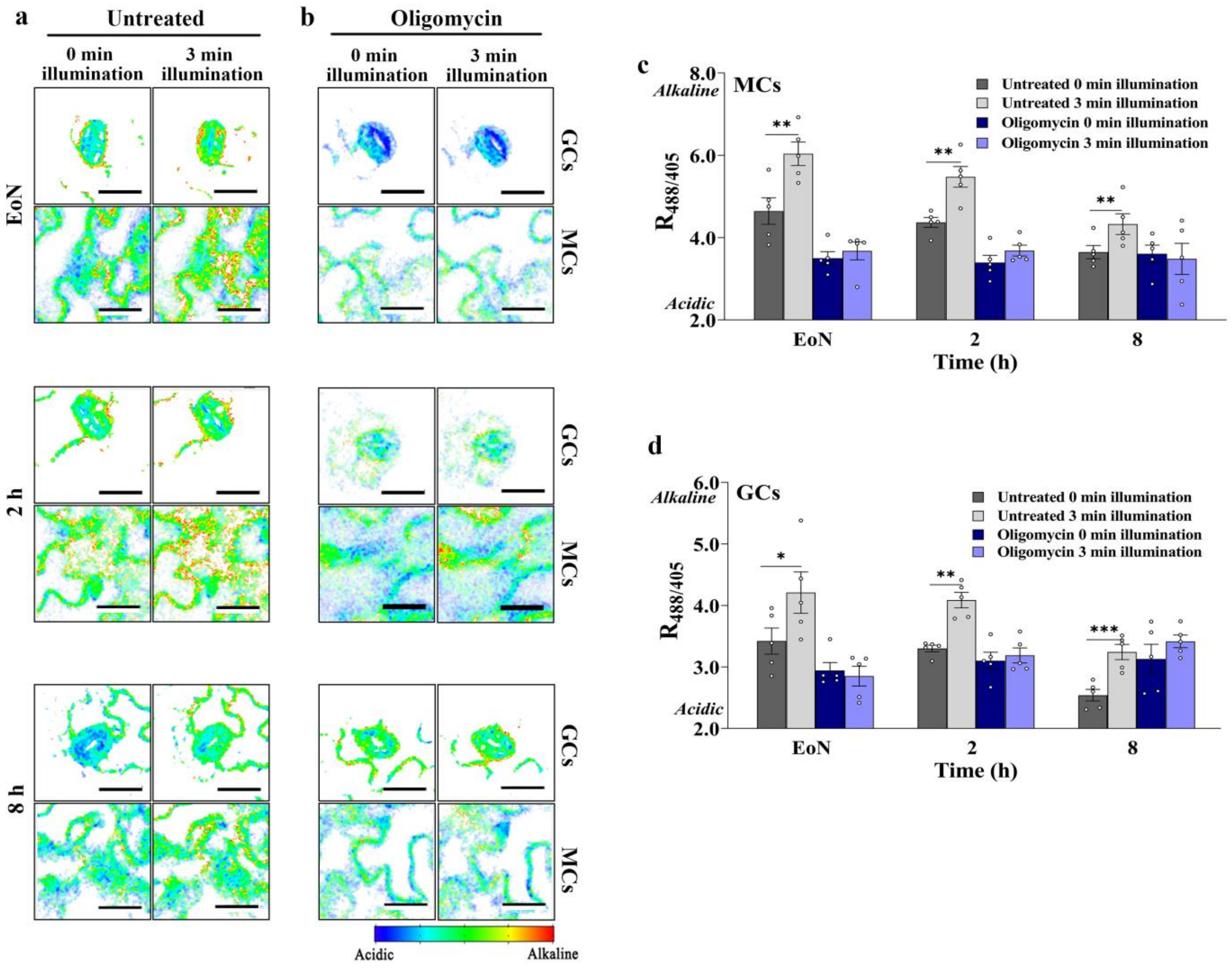

Supplementary Figure 3. Ratiometric images of cytosolic cpYFP expressed in mesophyll cells and guard cells before and after 3 min of illumination.

a–b, Mature leaves (4th leaf) of 20- to 22-day-old plants expressing cytosolic cpYFP were imaged at different time points (EoN, 2 h and 8 h into the day) **a**, Without or **b**, with 0.01 mM oligomycin pre-treatment and illumination with 216 µmol m^{-2} s^{-1} white light for 3 min after the first image was captured at 0 min. The images are displayed in pseudo colours. Scale bars, 20 µm. **c-d,** Changes in cytosolic pH in mesophyll cells (MCs) and guard cells (GCs) of 20- to 22-day-old plants (4th leaf) transformed with TKTP-cpYFP (pH sensor) in response to white light illumination at 216 µmol m^{-2} s^{-1} for 180 s in presence or absence of 0.01 mM oligomycin. Asterisks (*) indicate significant statistical differences (*$P < 0.05$, **$P < 0.01$, ***$P < 0.001$) as determined by paired *t*-test, two-tailed ($n = 5$; mean ± SEM); p-values of panel c: MCs EoN = 0.01, MCs 2 h = 0.007, and MCs 8 h = 0.006; p-values of panel d: GCs EoN = 0.034, GCs 2 h = 0.002, and GCs 8 h = 0.8×10^{-4}. **EoN**, End of night. Source data are provided as a source data file.

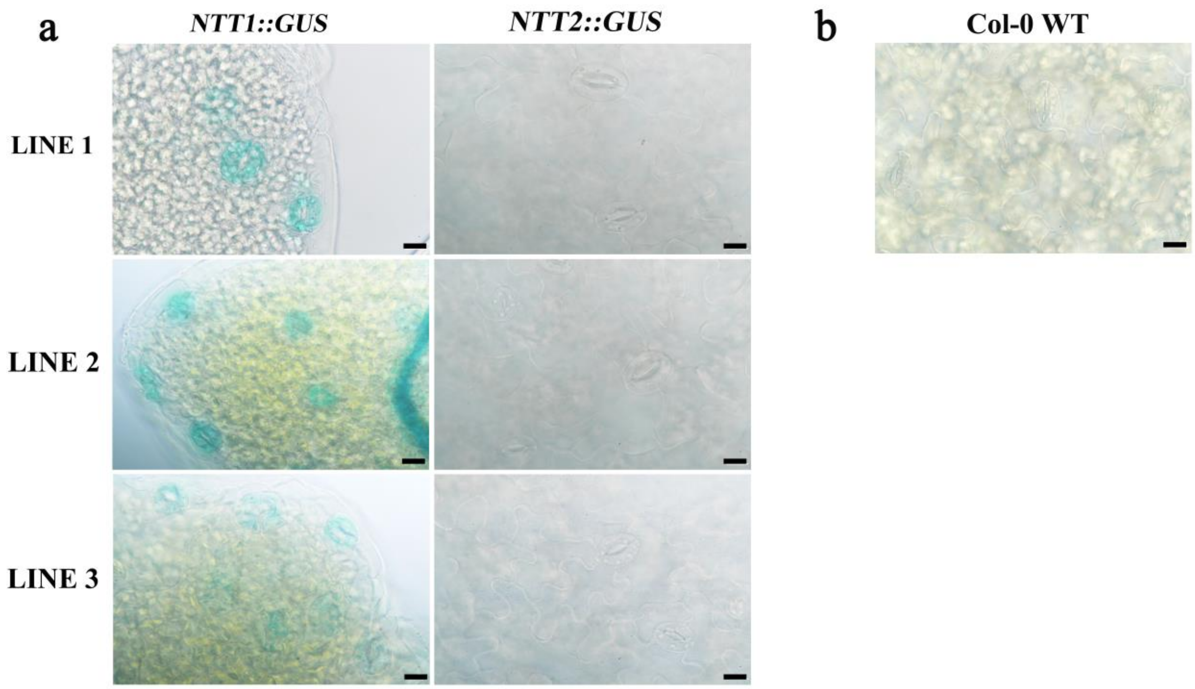

Supplementary Figure 4. Histochemical localization of *GUS* expression of *NTT* promoters in Arabidopsis seedling.
a, NTT1 promoter::GUS and NTT2 promoter::GUS activity in 21-day-old seedlings. **b,** Wild type (Col-0 WT) acts as control. This experiment was repeated three times independently. Scale bar, 10μm.

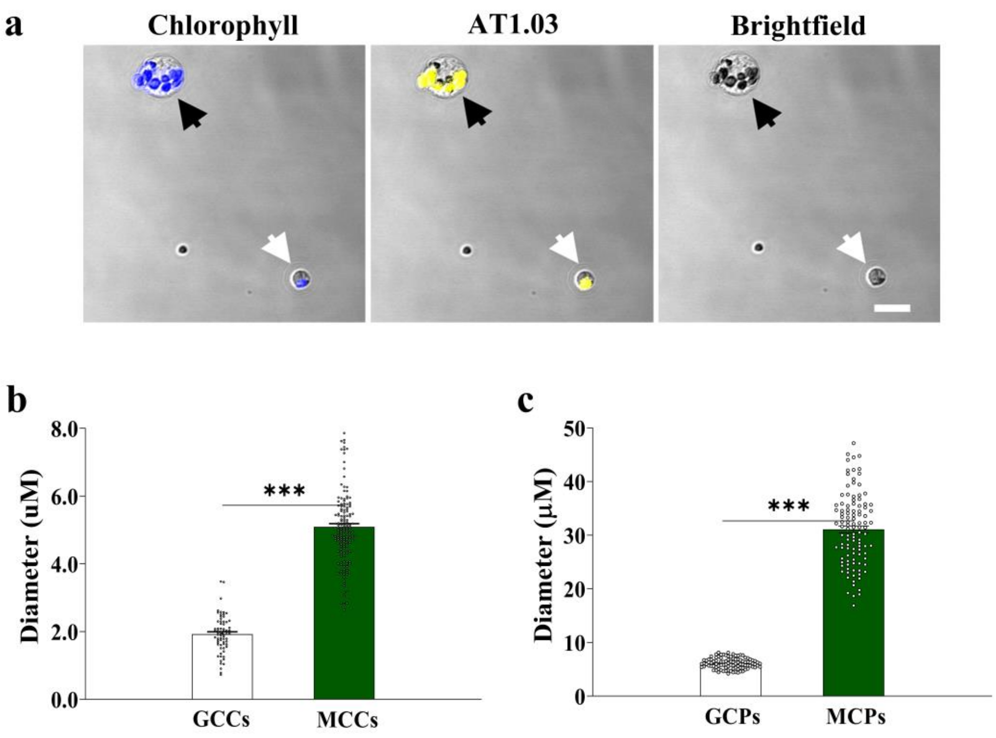

Supplementary Figure 5. Size comparison between protoplasts and chloroplasts isolated from guard cells and mesophyll cells.

a, Representative images of isolated guard cell protoplasts (GCPs, white arrow) and mesophyll cell protoplasts (MCPs, black arrow). Fluorescence images of chlorophyll fluorescence (Chlorophyll), Venus/CFP ratios (AT1.03), and transmitted light (Brightfield) are presented. Scale bar, 10 µm. **b**, Diameter of guard cell chloroplasts (GCCs) and mesophyll cell chloroplasts (GCCs; *n* = 68 and 163, respectively; mean ± SEM of two independent experiments). Asterisks indicate statistically significant differences (****P* < 0.001) between the diameters (µm) of chloroplasts of GCs and MCs and protoplasts of GCs and MCs, as determined by unpaired *t*-test, two-tailed (p-value of panel b: GCCs vs. MCCs = 3.3 x 10^{-74}. **c**, Diameter of GCPs and MCPs (*n* = 99 and 107, respectively; mean ± SEM of two independent experiments; p-value of panel c: GCPs vs. MCPs = 5.4 x 10^{-66}). Asterisks indicate statistically significant differences (****P* < 0.001) between the diameters (µm) of chloroplasts of GCs and MCs and protoplasts of GCs and MCs, as determined by unpaired *t*-test, two-tailed. Source data are provided as a source data file.

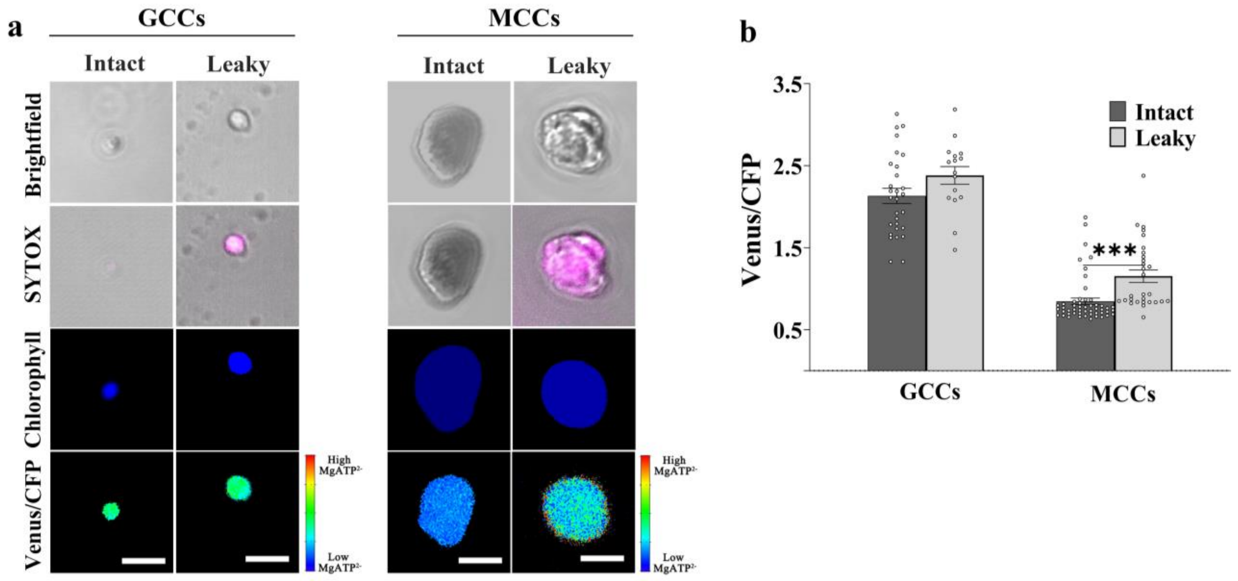

Supplementary Figure 6. Integrity of protoplasts and chloroplasts of mesophyll cells and guard cells.
a–b, Intact and leaky guard cell chloroplasts (GCC) and mesophyll cell chloroplasts (MCC) expressing TKTP-AT1.03. Leaky chloroplasts were stained with SYTOX™ orange and incubated with 5 mM ATP. Fluorescence images of transmitted light (brightfield), chlorophyll fluorescence (chlorophyll), SYTOX orange fluorescence (SYTOX), and Venus/CFP ratios (FRET) of chloroplasts are presented. FRET ratios and the corresponding SYTOX orange intensities of chloroplasts are displayed in the graph. The Asterisks indicate significant statistical differences (***$P < 0.01$) between intact and leaky chloroplasts, as determined by unpaired *t*-test, two-tailed (n =29, 16, 51 and 29, respectively; mean ± SEM) (p-value of panel b: MCCs = 0.001). Lower FRET ratio is expected in the chloroplasts with SYTOX orange stain as the stain absorbs the FRET emission at 526–545 nm. Scale bars, 5 µm. $MgATP^{2-}$ FRET ratios are represented by pseudo colour images, where higher ratios (red) correspond to higher $MgATP^{2-}$ levels. Source data are provided as a source data file.

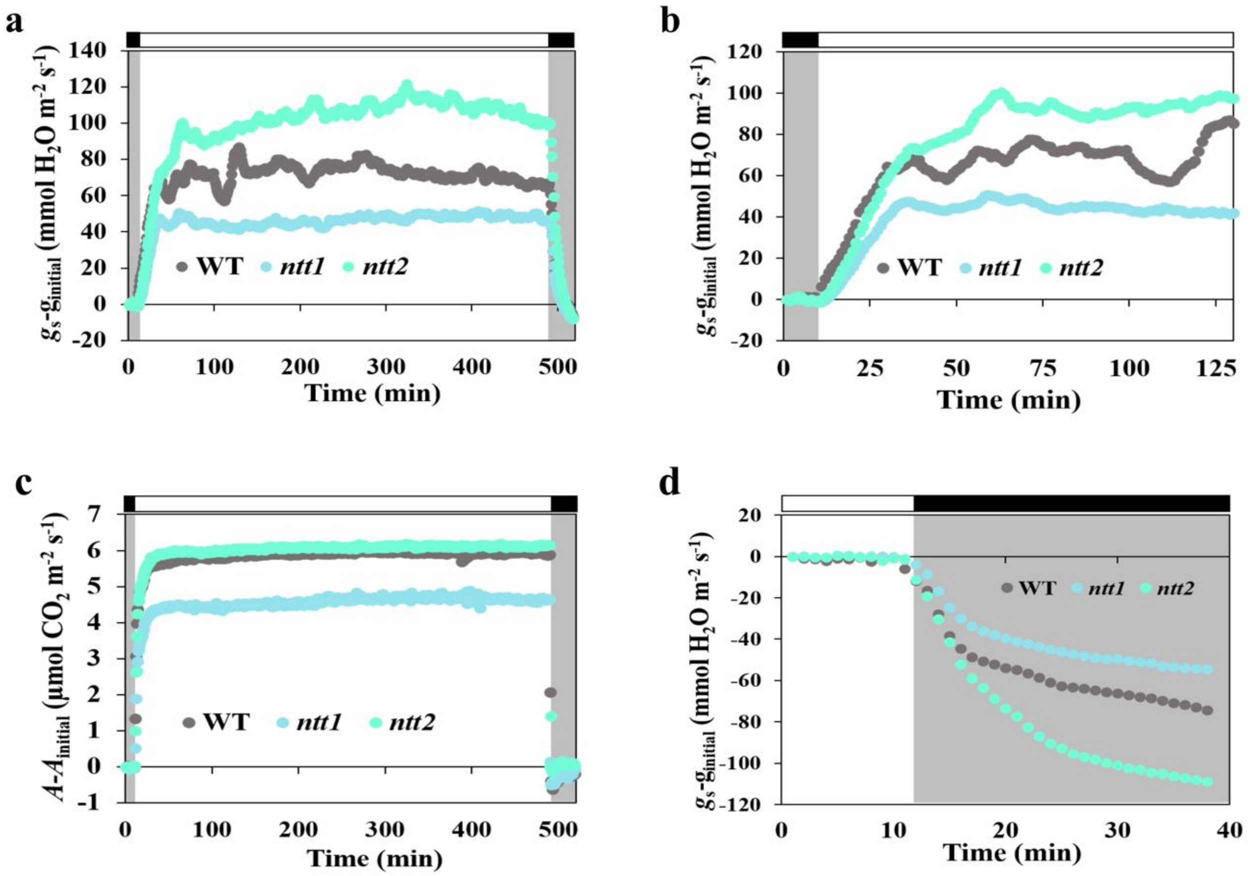

Supplementary Figure 7. Whole-plant recordings of gas exchange measurements of wild-type, *ntt1* and *ntt2* plants.

a, Normalized whole-plant recordings of changes in stomatal conductance (g_s) of wild-type, *ntt1* and *ntt*2 plants. Data shown are mean ± SEM; n = 3 per genotype. Plants have been illuminated with 150 µmol m^{-2} s^{-1} white light after end of night (EoN) under ambient-air CO_2 concentrations for 8 h. g_s values were normalized to values at EoN (0 = g_{initial}). **b**, Changes in $g_s - g_{\text{initial}}$ values in response to a shift from dark to light in wild-type, *ntt1* and *ntt2* plants. Data are replotted from normalized g_s plot. **c**, Normalized whole-plant recordings of changes in CO_2 assimilation (*A*) of wild-type, *ntt1* and *ntt2* plants. Data shown are mean ± SEM; n = 3 per genotype. Plants were illuminated with 150 µmol m^{-2} s^{-1} white light after EoN under ambient-air CO_2 concentrations for 8 h. *A* values were normalized to values at EoN (0 = A_{initial}). **d**, Changes in $g_s - g_{\text{initial}}$ values in response to a shift from light to dark in wild-type, *ntt1* and *ntt2* plants. Data are replotted from the normalized g_s plot. Data shown are mean ± SEM; *n* = 3 per genotype. Source data are provided as a source data file.

Supplementary Table 1. Primer sequences used for RT-qPCR

Gene	AGI code	Forward primer	Reverse primer	PCR efficiency	Reference
ACT2	At3g18780	CGTACAACCGGTATTGTGCT	GTAATCAGTAAGGTCACGTCCA	2.06	Horrer, Flütsch [1]
NTT1	At1g80300	TAAGTCGCTGGAGGGACAGT	TTCGTCCTGAGACACGACAG	2.07	Selinski, Konig [2]
NTT2	At1g15500	TTATGGGCGACTTCTCAACC	AAGGCAACACCGGTCAATAG	2.14	Selinski, Konig [2]

Supplementary Table 2. Primer sequences used for plasmid construction

Primer	Sequence (5'-3')	Reference
AtNTT1-Forward	gaccatgattacgccaagcttTGGACCTACATATGGGTTCGATT	Reiser, Linka [3]
AtNTT1-Reverse	ataagggactgaccacccgggCTCTCTATTTCACTCTCTCCCGCA	Reiser, Linka [3]
AtNTT2-Forward	gaccatgattacgccaagcttGGAAGAATCTGAAGTTTTGGAACC	Reiser, Linka [3]
AtNTT2-Reverse	ataagggactgaccacccgggCTCTCTATCTCTCACGTAGCACACTGA	Reiser, Linka [3]

References

1. Horrer D, *et al.* Blue light induces a distinct starch degradation pathway in guard cells for stomatal opening. *Curr Biol* **26**, 362-370 (2016).

2. Selinski J, *et al.* The plastid-localized NAD-dependent malate dehydrogenase is crucial for energy homeostasis in developing *Arabidopsis thaliana* seeds. *Molecular plant* **7**, 170-186 (2014).

3. Reiser J, Linka N, Lemke L, Jeblick W, Neuhaus HE. Molecular physiological analysis of the two plastidic ATP/ADP transporters from Arabidopsis. *Plant Physiol* **136**, 3524-3536 (2004).
